# COGNITIVE IMPAIRMENT AND SOCIAL CONNECTEDNESS: A DYADIC ANALYSIS OF OLDER COUPLES

**DOI:** 10.1093/geroni/igad104.0234

**Published:** 2023-12-21

**Authors:** Meiyi Li, Yiang Li

**Affiliations:** University of Wisconsin-Madison, Madison, Wisconsin, United States; University of Chicago, Chicago, Illinois, United States

## Abstract

Sociological research extensively identifies social connectedness as an important predictor of cognitive health. However, few examine how social connectedness will in turn be affected by cognitive health, especially in the dyadic context. Using couple data from Waves 2 and 3 of the National Social Life, Health, and Aging Project (NSHAP), this study investigates the gendered dyadic link between cognitive impairment and social connectedness. Results from the actor–partner interdependence models show that women’s cognitive impairments negatively affect their socialization. In terms of partner effects, women’s cognitive impairments make negative impacts on men’s number of friends and neighborhood participation. However, women’s social connectedness will not be affected by men’s cognitive impairment. Given the interdependence of older couples, this study points to the importance of understanding cognitive impairment in dyadic contexts. Results also call for dyadic interventions to prevent the negative effects caused by cognitive decline in late life.

